# Biomarkers of basal cell carcinoma resistance to methyl-aminolevulinate photodynamic therapy

**DOI:** 10.1371/journal.pone.0215537

**Published:** 2019-04-24

**Authors:** Tamara Gracia-Cazaña, Marta Mascaraque, Silvia Rocío Lucena, Jesús Vera-Álvarez, Salvador González, Ángeles Juarranz, Yolanda Gilaberte

**Affiliations:** 1 Department of Dermatology, Hospital de Barbastro, Huesca, Spain; 2 Departament of Biology, Universidad Autónoma de Madrid, Madrid, Spain; 3 Pathology Service, Hospital San Jorge, Huesca, Spain; 4 Department of Medicine and Medical Specialties, University of Alcala, Alcalá de Henares, Madrid, Spain; 5 Dermatology Service, Hospital Miguel Servet, Zaragoza, Spain; Columbia University Medical Center, UNITED STATES

## Abstract

**Background:**

Methyl-aminolevulinate photodynamic therapy (MAL-PDT) is an excellent option for the treatment of basal cell carcinoma (BCC). However, up to 25% of cases are resistant to this treatment modality.

**Objective:**

The aim of this study was to identify potential biomarkers of BCC response to MAL-PDT.

**Material and methods:**

Clinical, histological, and immunohistochemical (p53, Ki-67, CD-31, COX2, β-catenin, EGFR, and survivin) variables were analyzed in a retrospective study of consecutive BCC patients treated with MAL-PDT at the San Jorge Hospital, Huesca, Spain between January 2006 and December 2015. To deepen on these markers, the effects on p53 and cyclin D1 expression, *in vitro* response to MAL-PDT of 2 murine BCC cell lines (ASZ and BSZ), was also evaluated.

**Results:**

The retrospective study examined the response to MAL-PDT of 390 BCCs from 182 patients. The overall clinical response rate was 82.8%, with a mean follow-up time of 35.96 months (SD = 23.46). Immunohistochemistry revealed positive p53 in 84.6% of responders but only 15.4% of nonresponsive tumors (p = 0.011). Tumors with increased peripheral palisading of basal cell islands to immunostaining β-catenin responded poorly to PDT (p = 0.01). In line with our findings in patients, *in vitro* studies revealed a better response to PDT in the p53-positive ASZ cell line than the p53-negative BSZ cell line (p<0.01).

Multivariate analysis revealed that the following variables were significantly associated with response to PDT: age, nBCC, presence of peritumoral inflammatory infiltrate, and p53 immunopositivity. Patients with positive p53 immunostaining were 68.54 times more likely to achieve cure than p53-negative patients (CI95% 2.94–159.8)

**Conclusion:**

Our finding suggest that **c**ertain clinicopathological and immunohistochemical variables, particularly p53 expression, may serve as indicators of BCC response to MAL-PDT, and thus facilitate the selection of patients who are most likely to benefit from this therapy.

## Introduction

Methyl-aminolevulinate (MAL) photodynamic therapy (PDT) is an excellent option for the treatment of superficial (sBCC) and nodular (nBCC) basal cell carcinoma (strength of recommendation, A; quality of evidence, 1).[1] The clearance rate after 2 cycles of MAL-PDT is 91% after 3 months of follow-up, decreasing to 76% after 5 years of follow-up.[1] However, despite good response rates, primary or acquired resistance means that some tumors do not respond to treatment.[2]

Treatment resistance contributes to tumor progression and is associated with a worse prognosis. While resistance to chemotherapy and radiotherapy has been well-studied,[3] PDT resistance has received less research attention.[4,5] Common mechanisms involving extrinsic and intracellular factors may underlie resistance to PDT and other antitumoral therapies. Extrinsic factors include those pertaining to the tumor vasculature and stroma.[5] Intracellular alterations also may contribute to poor treatment responses to PDT.[4] Intracellular mechanisms implicated in PDT resistance include differences in the incorporation and expulsion rates of drugs; alterations in intracellular transport; loss of drug activity; increases in drug inactivation; and, in particular, the mutation and/or activation of certain genes implicated in BCC formation or altered following treatment.[6]

In this study, we sought to characterize clinical, histological, and molecular factors implicated in BCC response to MAL-PDT. To this end, we analyzed skin samples from BCC patients treated with MAL-PDT and studied the effects of MAL-PDT in 2 representative murine BCC cell lines, ASZ001 (ASZ) and BSZ2 (BSZ).[7]

## Patients and methods

### Design

In this retrospective observational study we analyzed samples from all consecutive patients who were clinically assisted by dermoscopy, and/or biopsy histologically diagnosed with BCC and treated with MAL-PDT between January 2006 and December 2015 at the Dermatology Service of San Jorge Hospital (Huesca, Spain). Histological samples were archived by the hospital’s Pathology Service. The following inclusion criteria were applied: clinical and dermoscopic diagnosis of BCC, a clinical follow-up period of >3 months, and available clinical and pathological records.

### MAL-PDT treatment

After lesion curettage or debulking, patients had received PDT with MAL cream (160 mg/g of Metvix; Galderma, France) following the standard procedure.[8] In all cases, the cream had been applied and incubated for 3 hours with occlusion, and subsequently exposed to 37 J/cm^2^ of illumination with a coherent, monochromatic red light source with a diode system (630 nm, Aktilite lamp; PhotoCure ASA, Oslo, Norway). The protocol was 2 sessions one week apart, each case was evaluated individually.

### Clinical variables

The clinical records of all patients were reviewed and data gathered for the following variables: BCC subtype (nBCC or sBCC), age at onset, sex, phototype (Fitzpatrick scale I–VI), and tumor size and location.

### Response-related variables

Clinical response was evaluated at the end of patient follow-up assisted with dermatoscopy and biopsy was performed in 63 cases. All patients were followed-up every 3 months for the first year after treatment and subsequently every 6 months up to a maximum of 6 years.

### Histological variables

Hematoxylin-eosin-stained sections were examined using an Olympus BX61 microscope (Olympus, PA, USA) coupled to a DP50 CCD digital camera (Olympus Optical Co. Ltd, Tokyo, Japan).

The following variables were evaluated: tumor thickness; histological subtype (superficial or nodular); peritumoral stroma (loose, dense or mucinous); presence/absence of elastosis; presence/absence of necrosis and/or ulceration; loss/enhancement peripheral palisading; and presence of peritumoral inflammatory infiltrate greater than 50% of the tumor area and vascularization.

### Immunohistochemical variables

The expression of biological markers implicated in PDT resistance, based on previous findings by our group,[4] was also evaluated using monoclonal antibodies against a range of proteins (S1 Table).

Sections were immunostained (TechMate 500, BioTech Solutions, Dako, Denmark) and then incubated with a detection kit (Chemate, code K4001, Dako) according to manufacturer’s recommendations. Immunostaining was visualized using 3-amino-9-ethylcarbazole chromogen solution (Dako). Heat-induced epitope retrieval was achieved using a pressure cooker. Representative sections and positive and negative controls were examined.[4]

For immunohistochemical evaluation of p53 and Ki-67 expression the tumor area with the highest levels of immunoexpression “hot spots” was identified and the percentage of cells with nuclear positivity in a high-power field (400×) was estimated. Expression of EGFR, survivin, β-catenin, and COX-2 was semiquantitatively assessed by classifying expression intensity as follows: 0, absence of staining; 1, mild staining (0–33% tumoral cell staining); 2, moderate staining (34–66% tumoral cell staining); and 3, intense staining (67–100% tumoral cell staining). For evaluation of CD-31 expression, vessels within the tumor with the highest levels of CD-31 expression were selected (“hot spot”) was identified and the number of CD-31-positive vessels in a high-power field (400×) was quantified. β-catenin immunostaining was categorized by expression intensity as well as reinforcement of peripheral palisading or not, while survivin expression was defined as focal or diffuse and expression intensity. All samples were simultaneously evaluated by 2 pathologists who were blind to sample identity.

### In vitro studies

#### Cell cultures

*In vitro* studies were performed using cell lines obtained from BCCs induced in a *ptch1*^*+/-*^ mouse exposed to UV irradiation (ASZ001, ASZ) and in a *ptch1*^*+/-*^, K14CreER2/+; p53fl/fl mouse exposed to γ radiation (BSZ2, BSZ).[7] Cells were grown in DMEM (Dulbecco’s modified Eagle’s medium) supplemented with 10% (v/v) fetal bovine serum and 1% antibiotic (penicillin, 10,000 units/ml; streptomycin 10,000 mg/ml), all from Thermo Fisher Scientific Inc. Cells were cultured under standard conditions (95% humidity, 5% CO_2_, 37°C) and propagated by trypsinization with 1 mM EDTA/0.25% trypsin (w/v).

#### Photodynamic treatment and MTT assay

Cells were grown in 24-well plates and incubated for 5 h with 0.3 mM MAL (Sigma-Aldrich, St. Louis, MO) in serum-free DMEM. Next, cells were irradiated at intensities of 0.45 J/cm^2^ to 2.25 J/cm^2^ using a monochromatic light source (635 nm ± 17 nm) with a multi-LED system. Control samples were subjected to identical conditions in the absence of irradiation and/or MAL.

#### MTT assay

Cell viability 24 h after photodynamic treatment was determined using the MTT (3-[4,5-dimethylthiazol-2-yl]-2,5-diphenyltetrazoliumbromide) assay. MTT solution (50 μg/ml) was added to cell cultures, which were then and incubated at 37°C for 3 h. After incubation, the formazan precipitate was dissolved with dimethylsulfoxide (DMSO, Panreac) and optical density was measured using a SpectraFluor (Tecan) plate reader (542 nm). Cellular toxicity was expressed as the number of surviving cells relative to the number of non-treated control cells.

#### Indirect immunofluorescence (IF)

For immunodetection of p53, β-catenin, and cyclin D1, cells were fixed in 3.7% formaldehyde (4°C) for 30 min, permeated with 0.1% Triton X-100 in PBS (v/v) for 30 min, and incubated with primary (1:100; Cell Signaling Technology, Inc. Danvers, MA) and secondary (1:250; Life Technologies, Eugene, Or, USA) antibodies. All preparations were counterstained and mounted with ProLong-Gold with DAPI (Life Technologies, Eugene, Or, USA).

#### Western Blot (WB)

Cellular extracts were obtained with RIPA buffer with Triton, pH 7.4 (Bioworld), containing phosphatase (PhosSTOP EASYpack, Roche) and protease (complete ULTRA tablets Mini EDTA-free EASYpack, Roche) inhibitors, following the manufacturer’s instructions. Protein concentration was determined using the BCA Protein Assay Kit (Pierce). Cellular extracts were diluted in Laemmli buffer (Bio-Rad) and heated for 5 min at 98°C. Electrophoresis was performed using acrylamide/bisacrylamide gels in denaturing conditions (SDS-PAGE) using a Mini-PROTEAN cell. Western blotting onto PVDF membranes (Bio-Rad) was performed using a Transblot Turbo system (Bio-Rad). Membranes were incubated with a after blocking solution consisting of skimmed milk in 0.1% TBS-Tween 20, with primary antibodies (anti-p53 and anti-β-catenin), and peroxidase-conjugated secondary antibodies (Thermo Fisher), following the manufacturer’s instructions. Protein bands were visualized by chemiluminiscence (ECL Pl us Kit, Amersham) using the high resolution ChemiDocTR XRS+ system (Bio-Rad), and digitalized using Image Lab version 3.0.1 software (Bio-Rad).

#### Microscopy

Images were obtained using an epifluorescence microscope coupled to a DP70 CCD camera (Olympus BX-61) equipped with corresponding filter sets: UV (360–370 nm excitation filter UG-1); blue (450–490 nm excitation filter BP 490); and green (570–590 nm excitation filter 590 DM). Images were processed with Photoshop Extended CS5 12.0 (Adobe Systems Inc., Mountain View, CA, USA). Quantitative image analysis was performed using ImageJ 1.8 software (Wayne Rasband National Institutes of Health, Bethesda, MD, USA).

### Statistical analysis

Data are expressed as the mean and standard deviation (SD) and dichotomous variables as proportions. Associations between qualitative variables were assessed using the Chi-squared test or Fisher’s exact test. Given the small sample size, the Mann-Whitney U-test or Kruskal-Wallis test for paired data were used to evaluate associations between quantitative variables. Statistical significance was set at p <0.05. Variables for which a statistically significant association with the response to PDT was observed were included in a multivariate analysis performed using logistic regression. SPSS Statistics software (Version 19.0: IBM Corp, Armonk, NY) was used for all analyses.

### Ethical concerns

The present study was strictly observational and involved no change to the regular care regimen of participating patients. All data were fully anonymized before accesing them. The study protocol was approved by the Aragón Ethical Committee for Clinical Research (CEICA) (CP-CI PI15/0219) and is part of a FIS research project (PI15/00974) S1 Fig.

## Results

We retrospectively analyzed the response to MAL-PDT of 390 BCCs from 182 patients. The overall response rate was 82.8%, with a mean follow-up time of 35.96 months (SD = 23.46; range, 3 months to 6 years). In all cases the same light fluency (37 J/cm^2^) was used. The majority of patients (87.4%) underwent 2 PDT sessions.

### Clinical variables

The results are summarized in Table 1. The mean age of study participants was 72.82 years (SD = 13), and was higher in non-responders (74.36 years) versus responders (69.22 years) (p = 0.007). Sixty percent were male and 40% female. The mean (± SD) tumor size was 10.42 ± 7.75 mm. Lesion distribution was as follows: head and neck, 45.4%; extremities, 10.3%; trunk, 16%. A correlation between lesion location and MAL-PDT response was observed: the poorest response rate was seen for BCCs located on the nose (62.7%) and the best response rate for those located on the trunk (94.7%) (p = 0.003). Phototype data were only available for 70 cases; the cure rate was higher for lighter versus darker phototypes (89.1% vs 66.7%; p = 0.034).

**Table 1:** Summary of the clinical, histological, and immunohistochemical variables analyzed in BCC patients, stratified by treatment-responsive and -nonresponsive groups.

**Clinical Variable**		**Responder group**	**Non-responder group**	**P-value**
Age	years	69.22 (SD = 14.66)	74.36 (SD = 11.7)	0.007
Size*	mm	10.29 (SD = 7.87)	10.89 (SD = 7.35)	0.445
Sex	Male	195 (83.3%)	39 (16.7%)	0.742
	Female	128 (82.1%)	28 (17.9%)	
Phototype**	1–3	49 (89.1%)	6 (10.9%)	0.034
	4–6	10 (66.7%)	5 (33.3%)	
Location	Nose	37 (62.7%)	22 (37.3%)	0.003
	Head and neck (except nose)	148 (83.6%)	29 (16.4%)	
	Trunk	108 (94.7%)	6 (5.3%)	
	Extremities	30 (75%)	10 (25%)	
Tumor type	Superficial BCC	105 (93.8%)	7 (6.3%)	<0.001
	Nodular BCC	218 (78.4%)	60 (21.6%)	
Number of PDT sessions	1	33 (97.1%)	1 (2.9%)	0.001
	2	282 (82.7%)	59 (17.3%)	
	≥3	8 (53.3%)	7 (46.7%)	
**Histological variable**		**Responder group**	**Non-responder group**	**P-value**
Tumor thickness	mm	1.44 (SD = 1.11)	1.86 (SD = 0.92)	0.081
Intratumoral necrosis	Yes	9 (81.8%)	2 (18.2%)	1
	No	40 (76.9%)	12 (23.1%)	
Histological subtype	Superficial BCC	14 (87.5%)	2 (12.5%)	0.487
	Nodular BCC	35 (74.5%)	12 (25.5%)	
Peritumoral stroma	Loose	21 (80.8%)	5 (19.2%)	0.369
	Dense	28 (77.8%)	8 (22.2%)	
	Mucinous	0 (0%)	1 (100%)	
Loss of palisading	Yes	2 (100%)	0 (0%)	1
	No	47 (77%)	14 (23%)	
Increased vascularization	Yes	12 (85.7%)	2 (14.3%)	0.716
	No	37 (75.5%)	12 (24.5%)	
Elastosis	Yes	16 (78.3%)	5 (21.7%)	0.861
	No	26 (76.3%)	9 (23.7%)	
Ulceration	Yes	11 (68.8%)	5 (31.3%)	0.315
	No	38 (80.9%)	9 (19.1%)	
Inflammatory infiltrate	Yes	36 (85.7%)	6 (14.3%)	0.032
	No	13 (61.9%)	8 (38.1%)	
**Immunohistochemical variable**		**Responder group**	**Non-responder group**	**P-value**
CD31	Vessels in a high-power field (400×)	11 (SD = 8.48)	9,78(SD = 6.93)	0.626
P53	Positive	44 (84.6%)	8 (15.4%)	0.011
	Negative	5 (45.5%)	6 (54.5%)	
	Mean (SD)	34.39 (SD = 34.34)	22.93 (SD = 29.34)	0.261
Ki-67	Positive	48 (77.4%)	14 (22.6%)	1
	Negative	1 (100%)	0 (0%)	
	Mean (SD)	31.71 (SD = 22.41)	30.57 (SD = 26.69)	0.872
COX-2	Positive	20 (69%)	9 (31%)	0.12
	Negative	29 (85.3%)	5 (14.7%)	
EGFR	Moderate to intense positive	30 (76.9%)	9 (23.1%)	0.835
	Mild positive-Negative	19 (79.2%)	5 (20.8%)	
β-catenin (Intensity)	Moderate to intense positive	33 (84.6%)	6 (15.4%)	0.096
	Mild positive-Negative	16 (66.7%)	8 (33.3%)	
β-catenin (Distribution)	Peripheral reinforcement of the islets	0 (0%)	3 (100%)	0.01
	Non peripheral reinforcement of the islets	48 (81.6%)	11 (18.6%)	
Survivin (Intensity)	Moderate to intense positive	30 (76.9%)	9 (23.1%)	0.903
	Mild positive-Negative	18 (78.3%)	5 (21.7%)	
Survivin (Distribution)	Focal	10 (83.3%)	2 (16.7%)	0.715
	Diffuse	37 (75.5%)	12 (24.5%)	

*Size and

**phototype data were only available for 214 and 70 BCCs, respectively.

Although the majority of patients (87.4%) underwent 2 PDT sessions, treatment response in these patients differ significantly from that of patients who underwent more than 2 sessions (p = 0.001).

Of the 390 BCCs included in the study, 278 were classified as nodular (nBCC) and 112 as superficial (sBCC). Significantly higher response rates were observed for sBCCs (93.8%) versus nBCCs (78.4%) (p <0.001).

### Histological variables

Of the patients that received MAL-PDT, 49 responders and 14 non-responders had previously undergone biopsy. The most common histological pattern was nBCC (74.6%). Higher response rates were observed for sBCCs versus nBCCs (87.5% vs 74.5%; p = 0.487).

**Fig 1:**
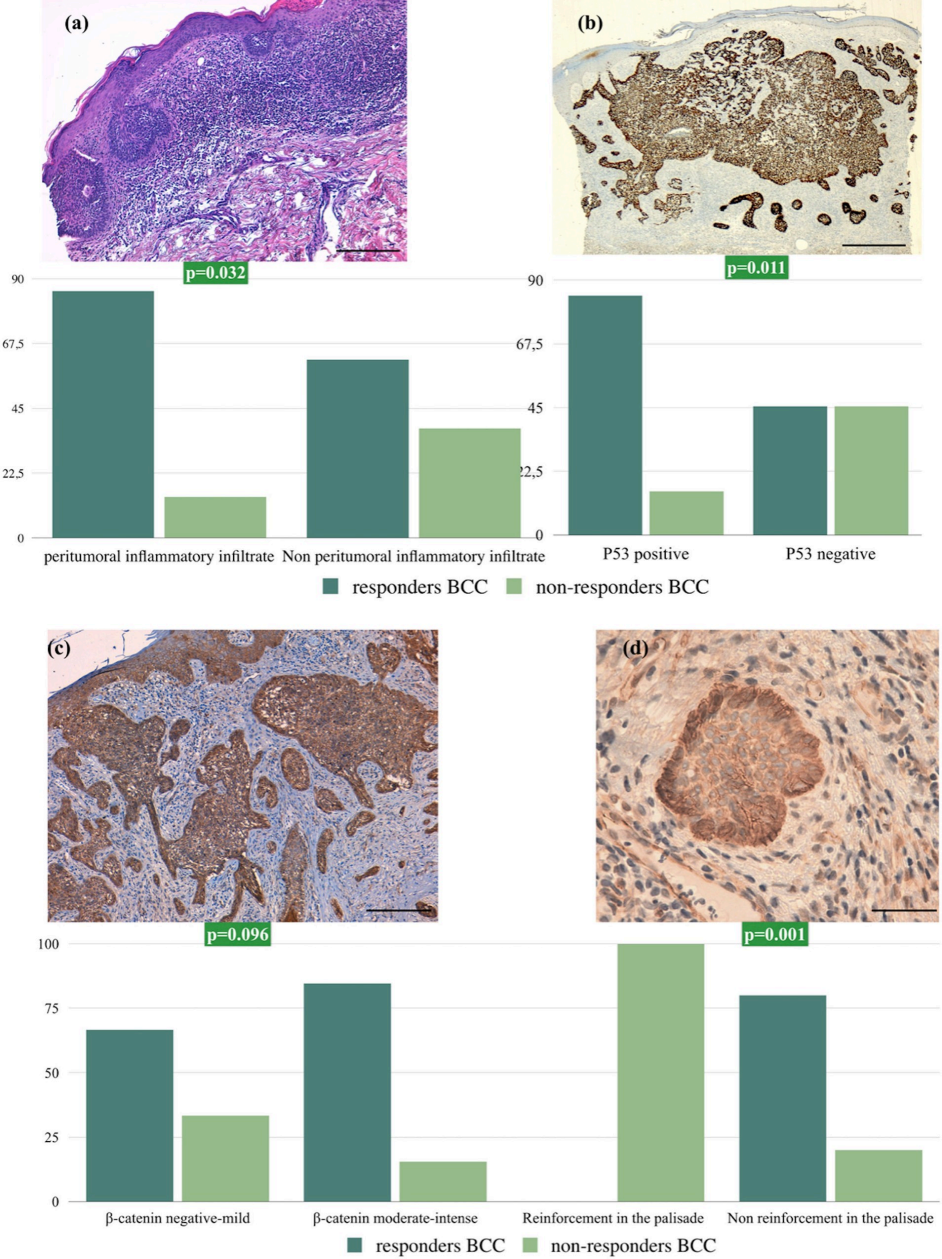
**(a)** Peritumoral inflammation surrounding basal cell carcinoma in a MAL-PDT-sensitive BCC (10×). **(b)** p53 immunostaining in a MAL-PDT responsive BCC: 97% of cells exhibit positive p53 immunostaining (5×). **(c)** Intense β-catenin immunostaining in a MAL-PDT-sensitive BCC (10×) and **(d)** enhanced peripheral palisading in a MAL-PDT-resistant BCC (40x). Bar charts depict levels of perilesional inflammatory infiltrate, p53 immunoexpression, and the intensity and distribution of β-catenin immunostaining in BCCs treated with MAL-PDT (responsive and nonresponsive). Scale bar: 200 μm (A and C), 500 μm (B) and 100 μm (D).

None of the histological variables were significantly associated with MAL-PDT response (Table 1), except for peritumoral inflammatory infiltrate: a higher response rate (85.7%) was observed for tumors with peritumoral inflammation (61.9%) (p = 0.032) (Fig 1A).

### Immunohistochemical variables

p53 and β-catenin were the only immunohistological variables for which a statistically significant association with the response to MAL-PDT was observed (Table 1). Positive p53 immunostaining was observed in 84.6% of responders, but only 15.4% of non-responders (p = 0.011) (Fig 1B). β-catenin immunostaining was moderate or intense in 84.6% of responders and in 33.3% of non-responders (p = 0.096). In 3 cases (4.83%), none of which responded to MAL-PDT (p = 0.01), a pattern of β-catenin staining with peripheral palisading reinforcement was observed (Fig 1C).

Multivariate analysis revealed that the following variables were significantly associated with response to PDT: age, nBCC, presence of peritumoral inflammatory infiltrate, and p53 immunopositivity. Patients with positive p53 immunostaining were 68.54 times more likely to achieve cure than p53-negative patients (CI95% 2.94–159.8) (Table 2).

**Table 2:** Results of the multiple logistic regression model showing variables significantly associated with treatment response: age, BCC subtype, presence of inflammatory infiltrate, and positive p53 immunostaining.

Coefficients	Estimation	Standard error	p-value	Odds Ratio (CI95%)
Age <63 years	0.263	0.096	0.006	1.3 (1.07–1.57)
Nodular BCC	-6.28	2.89	0.029	0.02 (0.0–0.53)
Inflammatory infiltrate	3.59	1.52	0.018	36.4 (1.84–716.5)
P53-positive	4.23	1.61	0.009	68.54 (2.94–159.8)

### In vitro studies

**Fig 2:**
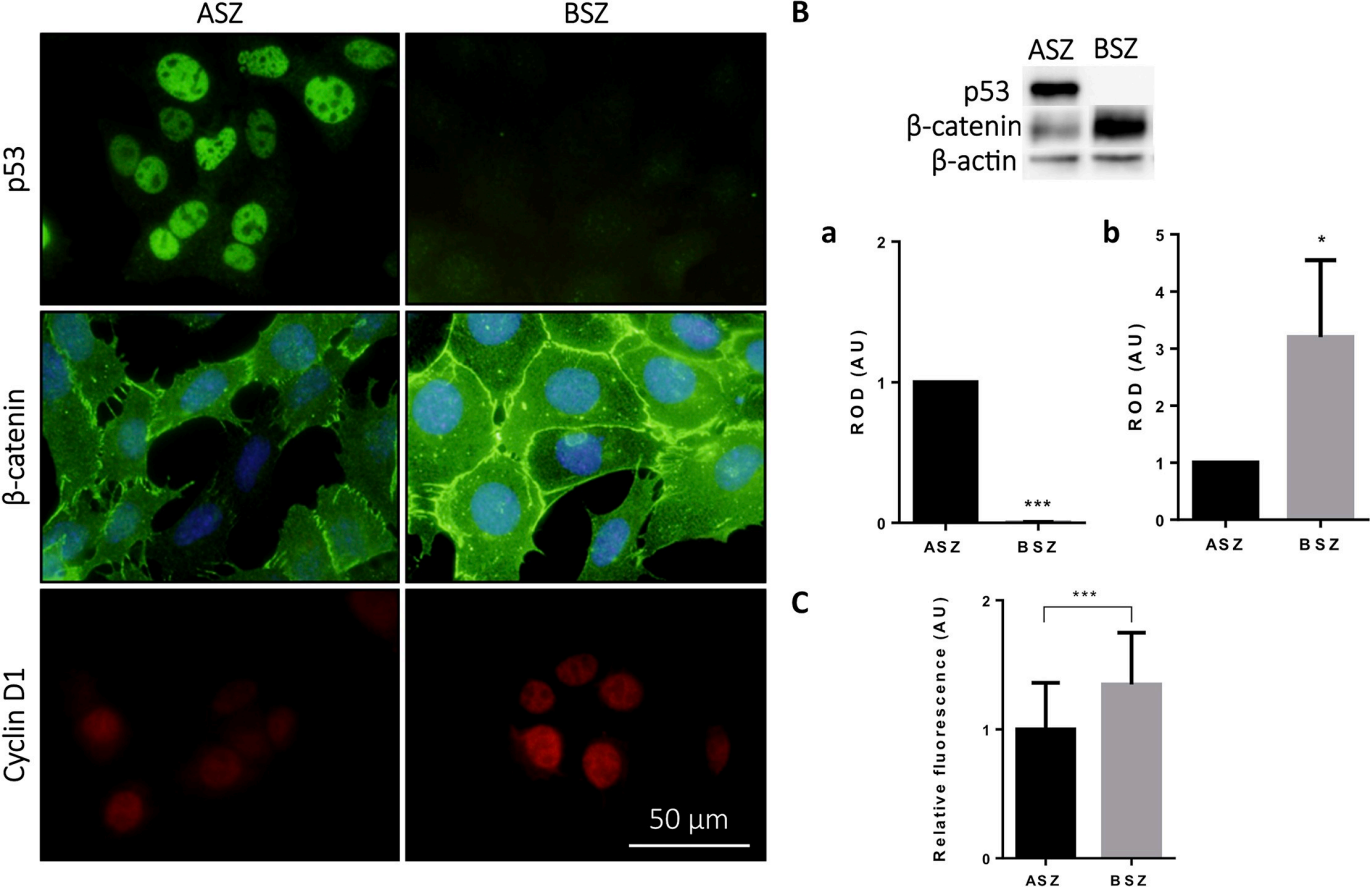
Expression of p53, β-catenin and ciclin D1 in BCC mice lines. (A) Expression pattern of p53, β-catenin and ciclin D1 on ASZ and BSZ cells by immunofluorescence. Scale bar: 20um. (B) Protein quantification by Western Blot of (a) p53 and (b) β-catenin. Load control: β-actin. (C) Relative fluorescence of cyclin d1 expression by immunofluorescence. *p< 0.05; ***p< 0.001.

As described above, negative p53 immunostaining and a specific pattern of β-catenin expression were associated with a poorer response to MAL-PDT in BCC patients. Next, we sought to corroborate this finding in 2 murine BCC cell lines: ASZ (p53-positive) and BSZ (p53-negative).^7^ First, using IF and WB (p <0.001 in both cases), we confirmed that p53 was expressed only in ASZ cells (Fig 2A and Fig 2B).

β-catenin expression was observed in both cell lines, primarily in the cell membrane, although diffuse cytoplasmic expression was also detected. The β-catenin signal was more intense in BSZ cells, in which higher levels of expression were confirmed by WB (p <0.05) (Fig 2B).

Levels of p53 and β-catenin expression were correlated with those of cyclin D1 (Fig 2A). In the p53-negative BSZ cell line, in which β-catenin expression was greatest, the mean fluorescence intensity of cyclin D1 was significantly higher than that observed in ASZ cells (p <0.001) (Fig 2C).

### Cell viability after photodynamic treatment

**Fig 3:**
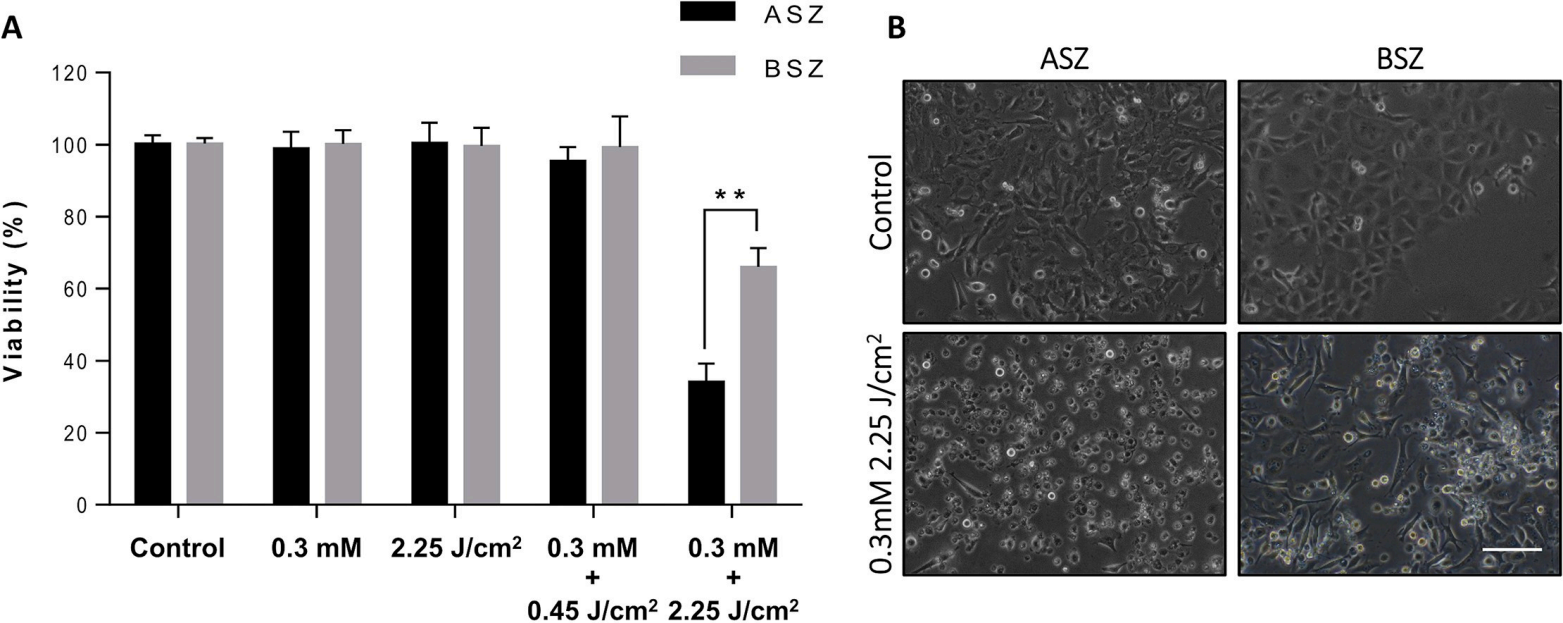
**(a)** Cell viability of ASZ and BSZ cells as determined by MTT assay in drug control (0.3 mM MAL), light control (2.25 J/cm^2^), and PDT (0.3 mM MAL + 0.5 J/cm^2^ or 0.3 mM MAL + 2.25 J/cm^2^) conditions. **p <0.01. **(b)** Photographs of ASZ and BSZ cells following PDT. Scale bar, 200 μm.

ASZ and BSZ cells were incubated with 0.3 mM MAL for 5 h and subsequently irradiated with different doses of red light. MTT assay revealed no effect on the viability of ASZ or BSZ cells of successive exposure to the 2 components of MAL-PDT (Fig 3A). PDT (MAL 0.3 mM; 2.25 J/cm^2^) resulted in decreases in cell viability to 35% (ASZ cells) and 66% (BSZ) of corresponding control levels (p <0.01). The difference in response was also seen in their cell morphology, after PDT; ASZ showed a major decrease of live cells and an increase of cellular damage than BSZ (Fig 3B).

## Discussion

In this study we retrospectively evaluated the effects of MAL-PDT on different clinical-pathological and molecular characteristics of BCC. Analysis of clinical variables revealed that the response of nBCCs was poorer than that of sBCCs. Other factors associated with a poorer response were location of the tumor in area H, older age, darker phototype, and a greater number of MAL-PDT sessions. The only histological variable associated with a poor response was the absence of peritumoral lymphocytic inflammatory infiltrate. Finally, we found that negative p53 immunoreactivity and a β-catenin pattern with peripheral reinforcement of islands of basaloid cells were associated with tumor resistance to PDT. These molecular findings were corroborated *in vitro* by IF and WB in BCC cell lines.

The rate of response to PDT was influenced by the histological subtype of BCC (87.5% for sBCC vs. 74.5% for nBCC). Previous studies have reported higher cure rates for sBCCs (82–100%) [9–11] than nBCCs (33–100%) [11–18] after PDT. This observation may be directly related to other parameters, such as tumor thickness. Morton *et al*. proposed that lesion thickness influences the response to topical PDT, setting a thickness limit of 2 mm.[19]

The effectiveness of PDT may be limited by other clinical and epidemiologic factors, including age and tumor location. According to our findings, the H area is the least suitable area for PDT, it can be explained because they are embryological fusion areas with a tendency to invade in depth or maybe the locations of lesions reflect accumulation of UV lesions and hence, status of mutations.[20] In our series of 472 tumors (BCC and Bowen disease) treated with MAL-PDT, we found that more advanced age was a predictor of a poor response. Supporting this view, Niessen *et al*. found that PDT was more effective in younger patients, and reported an age-associated decrease in the formation of PpIX after application of MAL or BF-200.[21] However, other authors have reported no such association between older age and a poorer treatment response or higher recurrence rate.[22]

Our results suggest that darker phototypes may be associated with a poorer response to MAL-PDT. This may be the result of competitive absorption of light by melanin in the basal layer of the epidermis, and a consequent decrease in the total amount of energy that reaches deeper dermal lesions.[23] It should be borne in mind that melanin is an endogenous antioxidant of the skin, and may scavenge reactive oxygen species produced during PDT, thus limiting treatment efficacy.[24]

We found that clinical outcome was not improved in patients who received more than 2 PDT sessions. Therefore instead of insisting it has been shown that the combination of PDT with other therapies increases the likelihood of success, diminishing resistance.[2]

We observed a significant correlation between the presence of intense inflammatory lymphocytic infiltrate and a better response to treatment, supporting previous findings by our group in squamous cell carcinomas (SCCs) treated with MAL-PDT.[4] In other tumor types, such as melanoma,[25] the presence of inflammatory infiltrate is a proposed prognostic factor, and may constitute an antitumor response from the host, contributing to or even enhancing the effect of PDT.[26]

Positive p53 immunoexpression was detected in 15.4% of MAL-PDT-resistant BCCs versus 84.6% of MAL-PDT-responsive BCCs, a difference that proved statistically significant. This finding was corroborated in the *in vitro* study, in which a better response to PDT was observed in the p53-positive (ASZ) than the p53-negative (BSZ) BCC cell line. We previously reported similar findings in Bowen's disease patients treated with MAL-PDT and in the SCC cell lines SCC-13 and A-431.[27] Furthermore, the findings of multiple *in vivo* studies suggest that p53 may play a role in the observed increase in PpIX levels and subsequent cell death with increased selective accumulation.[28–31]

β-catenin expression has been linked to tumor aggressiveness [32]: intranuclear β-catenin expression is correlated with increased tumor proliferation and aggressiveness, and is observed in the most aggressive subtypes. In BCCs that did not respond to MAL-PDT we identified a characteristic β-catenin immunostaining pattern at the advancing border with increased palisading. Ciurea *et al*.[32], El-Bahrawy *et al*.[33] and Oh *et al*.[34] reported that in BCCs with an infiltrative component β-catenin immunostaining is increased at the advancing border and at the periphery of nodules in more indolent variants, strongly supporting a role of β-catenin in BCC invasion. At cellular level, higher expression of total β-catenin was associated with the absence of p53 expression in BSZ cells, confirming the evidences in bibliography that link the absence of p53 with a higher expression of Wnt/β-catenin factors.[35–36]

Compared with ASZ cells, cyclin D1 expression was higher in BSZ cells (in which p53 is absent and β-catenin expression is increased). Higher levels of cyclin D1 expression have been associated with a poorer prognosis in breast, ovarian, and esophageal carcinomas.[36–38]

Limitations of the present study include the significant number of cases that were lost to follow-up before 6 years, precluding analysis of factors influencing the long-term response to MAL-PDT.

In conclusion, our study identifies several possible biomarkers or histological features indicative of a poor BCC response to MAL-PDT that could be used to select patients who will most benefit from this treatment modality. These include advanced age (>63 years), nBCC, absent p53 expression, β-catenin peripheral palisading of basal cell islands reinforcement, and the absence of peritumoral infiltrate.

## Supporting information

S1 Fig Ethics committee of Aragon approval and reference number. (PDF)

S1 Table Information about the antibodies used in the study. (DOCX)
