# Supplementary material for: Biomarkers of basal cell carcinoma resistance to methyl-aminolevulinate photodynamic therapy
Source: PLoS One. 2019 Apr 24;14(4):e0215537. doi: 10.1371/journal.pone.0215537 (PMC6481917; doi:10.1371/journal.pone.0215537)
Supplement: S1 Fig — (PDF) [file pone.0215537.s001.pdf]

GOBIERNO DE ARAGON
Departamento de Sanidad

Informe Dictamen Favorable
Proyecto Investigación Biomédica

C.P. - C.I. PI15/0219

9 de septiembre de 2015

Dña. María González Hinjos, Secretaria del CEIC Aragón (CEICA)

CERTIFICA

1º. Que el CEIC Aragón (CEICA) en su reunión del día 09/09/2015, Acta Nº 14/2015 ha evaluado la propuesta del investigador referida al estudio:

Título: Determinación de marcadores de resistencia a terapia fotodinámica en el tratamiento de cáncer cutáneo no melanoma y búsqueda de estrategias para superarla.

Investigador Principal: Yolanda Gilaberte Calzada. Hospital general san Jorge

Versión protocolo: Julio/2015
Versión hoja de información a los participantes y consentimiento informado: 15/07/2015

2º. Considera que

- El proyecto se plantea siguiendo los requisitos de la Ley 14/2007, de 3 de julio, de Investigación Biomédica y su realización es pertinente.
- Se cumplen los requisitos necesarios de idoneidad del protocolo en relación con los objetivos del estudio y están justificados los riesgos y molestias previsibles para el sujeto.
- Son adecuados tanto el procedimiento para obtener el consentimiento informado como la compensación prevista para los sujetos por daños que pudieran derivarse de su participación en el estudio.
- El alcance de las compensaciones económicas previstas no interfiere con el respeto a los postulados éticos.
- La capacidad de los Investigadores y los medios disponibles son apropiados para llevar a cabo el estudio.

3º. Por lo que este CEIC emite **DICTAMEN FAVORABLE a la realización del proyecto.**

Lo que firmo en Zaragoza, a 9 de septiembre de 2015

Fdo:

GOBIERNO DE ARAGON
COMITÉ ÉTICO DE INVESTIGACIÓN CLÍNICA

Dña. María González Hinjos
Secretaria del CEIC Aragón (CEICA)

Tel. 976 71 48 57 Fax. 976 71 55 54 Correo electrónico mgonzalezh.ceic@aragon.es
