# Supplementary material for: Biomarkers of basal cell carcinoma resistance to methyl-aminolevulinate photodynamic therapy
Source: PLoS One. 2019 Apr 24;14(4):e0215537. doi: 10.1371/journal.pone.0215537 (PMC6481917; doi:10.1371/journal.pone.0215537)
Supplement: S1 Table — (DOCX) [file pone.0215537.s002.docx]

| Antibody | Commercial brand | Clone | Dilution | Monoclonal Antibody | Antigen unmasking | Staining |
| --- | --- | --- | --- | --- | --- | --- |
| Ki-67 | Ventana Medical System | 30-9 | Prediluted | Rabbit | Citrate CC1 pH:6 | Nucleus |
| P53 | Ventana Medical System | DO-7 | Prediluted | Mouse | Citrate CC1 pH:6 | Nucleus |
| CD-31 | Cell Marque Corporation | JC70 | Prediluted | Mouse | Citrate CC1 pH:6 | Cytoplasm and/or membrane |
| COX-2 | Cell Marque Corporation | SP21 | Prediluted | Rabbit | Citrate CC1 pH:6 | Cytoplasm |
| EGFR | Ventana Medical System | 3C6 | Prediluted | Mouse | Citrate CC1 pH:6 | Cytoplasm and/or membrane |
| β-Catenin | BD Biosciences | 610153 | 1/100 | Mouse | Citrate CC1 pH:6 | cytoplasm and / or nucleus and/or membrane |
| Survivin | Abcam | ab134170 | 1/400 | Rabbit | Citrate CC1 pH:6 | cytoplasm and / or nucleus |
